# Gut microbial communities in chronic rhinosinusitis patients in response to 1,8-Cineol treatment

**DOI:** 10.1016/j.crmicr.2025.100442

**Published:** 2025-07-22

**Authors:** Simon Graspeuntner, Mathias Heidemann, Stephanie Jeschke, Mariia Lupatsii, Zuzana Penxova, Sven Künzel, Barbara Wollenberg, Anke Leichtle, Michael Ploch, Karl-Ludwig Bruchhage, Ralph Pries

**Affiliations:** a Department of Infectious Diseases and Microbiology, University of Lübeck, Lübeck, Germany; b Medical Clinic III, University of Lübeck, Lübeck, Germany; c Department of Otorhinolaryngology, University of Lübeck, Lübeck, Germany; d Max-Planck Institute for Evolutionary Biology, Plön, Germany; e Department of Otorhinolaryngology, Technical University of Munich, München, Germany; f MCM Klosterfrau Vertriebsgesellschaft mbH Cologne, Köln, Germany

**Keywords:** Chronic rhinosinusitis, Nasal polyps, Gut microbiota, Inflammation, 1,8-Cineol

## Abstract

**Background:**

Chronic rhinosinusitis (CRS) is a widespread inflammatory disease of the paranasal sinuses and the standard treatment includes surgery and the administration of corticosteroids or antibiotics. Antibiotics are well known to influence the microbial composition of the gut, including a reduced bacterial diversity and an increased antibiotic resistance. This underlines the need for alternative treatment approaches, especially regarding vulnerable patients such as children. Therefore, we evaluated the intestinal microbiota of CRS patients regarding plant-based anti-inflammatory monoterpene 1,8-Cineol, which is administered as the clinically approved drug Soledum® for the therapy of different inflammatory airway diseases including CRS.

**Methods:**

Microbiome analysis from stool samples was performed from 31 CRS patients before and after 1,8-Cineol administration using 16S rRNA gene next generation sequencing using dissimilarity-based and taxonomic approaches.

**Results:**

Data revealed elevated abundances of specific taxa including *Bacteroides* and *Faecalibacterium* as part of the physiologic gut microbial community and no overall disturbances in response to 1,8-Cineol treatment. Of note, abundances of intestinal *Akkermansia muciniphila* tend to increase during the course of 1,8-Cineol administration.

**Conclusion:**

Our data recommend 1,8-Cineol as a valuable addition to the current standard treatment of inflammatory diseases, without running the risk of profound changes of the gut microbial community.

## Hightlights

• Chronic rhinosinusitis (CRS) patients reveal a dysbacteriosis of the nasal microbiota and increased levels of bacterial pathogens such as *Staphylococcus aureus,* which is suggested to trigger the development of nasal polyps.
• Common treatment options for chronic rhinosinusitis patients include systemic antibiotics, which are well known to influence the microbial composition of the gut, including a decrease in bacterial diversity and an increase in antibiotic resistance.
• Plant-based anti-inflammatory monoterpene 1,8-Cineol is commonly used as the clinically approved drug Soledum® for the treatment of inflammatory airway diseases including CRS replacing need for antibiotic therapy.
• Anti-inflammatory 1,8-Cineol does not cause overall disturbances of the physiologic gut microbial community, but promotes increased abundances of intestinal *Akkermansia muciniphila*, which has gained increasing attention as a promising probiotic bacterial species.
• Thus, 1,8-Cineol outcompetes antibiotic therapy by the means of lack of negative side effects on the gut microbiome, thereby promising increased patient´s wellbeing and reduced development of antimicrobial resistance following treatment.

## Graphical abstract

**Figure:**
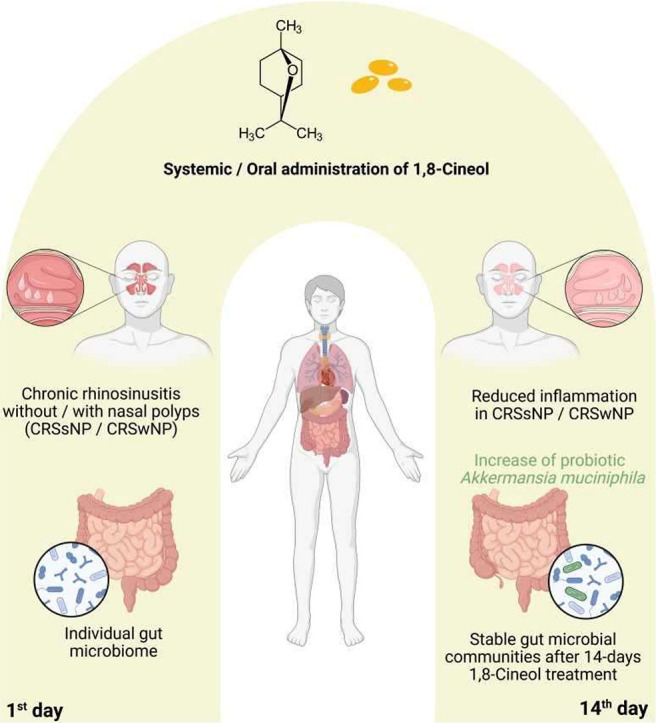

## Introduction

Chronic rhinosinusitis (CRS) is an inflammatory sinus disease that affects approximately 10 % of the European population and can occur without (CRSsNP) or with the development of nasal polyps (CRSwNP) (Hastan et al., 2011; Dietz de Loos et al., 2019). Several environmental factors such as air pollution or cigarette smoking and individual immune barrier dysfunctions were associated with the development of this disease (Fokkens et al., 2020; Pezato et al., 2012; Soyka et al., 2012). Furthermore, CRSwNP patients revealed a dysbacteriosis of the nasal microbiota and increased presence of different bacterial pathogens such as *Staphylococcus aureus* were suggested to trigger the development of nasal polyps (Boase et al., 2013; Tantilipikorn et al., 2012; Chalermwatanachai et al., 2018).

Standard treatment options for chronic rhinosinusitis patients comprise systemic or topic corticosteroids or antibiotics as well as surgery (Kucuksezer et al., 2018). In addition, different biologicals targeting Immunoglobulin E (IgE), Interleukin-5 (IL-5), IL-4 or IL-13 have been introduced into clinical use in the recent years (Lyly et al., 2020). It is well established, that the gut microbiota is closely associated with the course of different inflammatory diseases and that bacterial metabolic products from the gut are able to reach and influence distant organs via the systemic circulation (van Olst et al., 2021; Pu et al., 2021; Ke et al., 2021; Kociszewska and Vlajkovic, 2022). Furthermore, the gut microbiota is strongly perturbed by different factors including antibiotics, which can lead to a reduced microbial diversity and the development of antibiotic-resistant bacteria (Patangia et al., 2022). Accordingly, antibiotic-induced changes of the microbial composition are well known to be associated with different infections and autoimmune diseases of the gastrointestinal tract (Song et al., 2021; Fishbein et al., 2023).

Therefore, alternative anti-inflammatory treatment approaches are needed to reduce these adverse effects of antibiotics on microbiota and health, especially with regard to vulnerable patients such as children, multimorbid patients or people with known antbiotic resistances. In this context, the natural anti-inflammatory monoterpene 1,8-Cineol is commonly used as the clinically approved drug Soledum® for the treatment of different chronic and acute airway diseases including CRS (Pries et al., 2023; Juergens, 2014). Recent investigations on CRSwNP patients data revealed a wide spread systemic distribution of administered 1,8-Cineol in the human body via the gut and the blood stream, from where it is expelled through the lungs and the respiratory tract along the mucosal tissues (MacKenzie et al., 2023).

1,8-Cineol is the major bacteriostatic component of eucalyptus leaf essential oils and has also been shown to reduce secretion levels of pro-inflammatory mediators from different immune cells including monocytes and lymphocytes (Juergens et al., 1998; Ocana and Reglero, 2012; Juergens et al., 2004). Interestingly, the use of 1,8-Cineol as a feed supplement altered the ratio of the gut microbiota in chickens with increased proportions of *Lactobacillus* and *Escherichia*, whereas the proportion of *Salmonella* decreased (Jiang et al., 2020). Analogously, it was observed that patients suffering from chronic otitis media (COM) revealed reduced appearances of pro-inflammatory bacteria species Pseudomonas aeruginosa and Proteus mirabilis in ear samples (Leichtle et al., 2024). Furthermore, intestinal Prevotella copri abundance was associated with therapy response and an improved clinical outcome in certain individuals (Leichtle et al., 2024). Given the aforementioned concerns about antibiotic treatment in CRS, we emphasize the importance of understanding whether - and if so, how - 1,8-Cineol affects the gut microbial composition, and consequently, its impact on gut health. However, the influence of 1,8-Cineol on the intestinal microbiota composition in CRSwNP patients has not yet been investigated.

In this study, we therefore aim to comprehensively evaluate the dynamics of possible gut microbial changes in CRSwNP patients in response to a 14-day administration of 1,8-Cineol to improve our understanding of the holistic therapeutic effect of 1,8-Cineol in acute and chronic inflammations.

## Materials and methods

### Ethics statement

Patients were examined and treated surgically after written informed consent at the Department of Otorhinolaryngology, University Hospital Schleswig-Holstein, Campus Luebeck. The presented investigations were approved by the local ethics committee of the University of Luebeck (approval number 18–322) and performed according to the WMA Declaration of Helsinki.

### CRSwNP patients and 1,8-Cineol administration

CRS patients were included into the study after physical examination, nasal endoscopy, and computed tomography (CT) scan of the sinuses. We investigated the intestinal microbial composition of 31 CRSwNP patients (10 females/21 males; mean age of 56) prior and after 14 days of 1,8-Cineol treatment.

1,8-Cineol (CNL-1976®) was administered as the clinically approved drug Soledum® Kapseln forte (capsules) (Cassella-med GmbH & Co. KG, Cologne, Germany) over 14 days (3 × 200 mg 1,8-Cineol/day) without any antibiotics during this time.

### Gut microbiome analysis

An analysis of the microbial community was conducted on a set of 31 pairs of pre- and post- 1,8 Cineol samples. Stool samples were collected in Stool Collection Tubes with DNA Stabilizer (Invitek Molecular GmbH, Berlin, Germany) and PSP® Spin Stool DNA Plus Kit (Invitek Molecular GmbH, Berlin, Germany) was used for further processing.

DNA amplification was performed by targeting the V3/V4 hypervariable region of 16S rRNA gene as described before (Graspeuntner et al., 2018). The following setup was applied: initial denaturation at 98 °C for 30 s followed by 30 cycles of 98 °C for 9 s, 55 °C for 60 s and 72 °C for 90 s, finalized with elongation for 10 min at 72 °C. Amplicon concentrations were analyzed on the basis of band intensity in 2 % agarose gel electrophoresis in comparison to reference bands (GeneRuler 100 bp DNA Ladder;Thermo Fischer Scientific, Waltham, USA). Equimolar amplicon concentrations were pooled and purified from a gel via MinElute® Gel Extraction Kit (Qiagen GmbH, Hilden, Germany). The final library concentration was estimated using NEBNext® Library Quantification Kit for Illumina® (New England Biolabs, Ipswich, Massachusetts, USA) and next generation sequencing was conducted using the MiSeq® Reagent Kit v3 (600 cycles) (Illumina®, San Diego, California, USA). PhiX Control Library v3 (Illuminaࣨ, San Diego, California, USA) was used to control for sequencing quality, cluster generation and alignment. Raw sequences were processed via mothur 1.44.1 (Schloss et al., 2009) with alignment and taxonomic assignment based on the EZBioCloud reference data base (Chalita et al., 2024). Chimeric sequences were filtered using the VSEARCH algorithm (Rognes et al., 2016) and operation taxonomic unit allocation was performed with a cutoff level of 0.03.

Microbial community analysis was conducted using R (version 4.0.1). Heatmaps were created using BoutrosLab.plotting.general package (P'ng et al., 2019). Relative abundances of microbial taxa were compared via pairwise Wilcoxon rank-sum test with corrections for false detection rate in the stats package while alpha diversity measures were compared using Wilcoxon rank-sum test. Analysis of beta diversity was performed via Principal Coordinates Analysis using the labdsv package (Jager et al., 2011) and permutational multivariate analysis of variance via distance matrices (vegan package) was carried out to evaluate the degree of dissimilarity (Tikhonov et al., 2020). Indicator species were analyzed using MaAsLin2 within R (Mallick et al., 2021).

## Results

### Gut microbial composition upon 1,8 Cineol administration

**Fig. 1:**
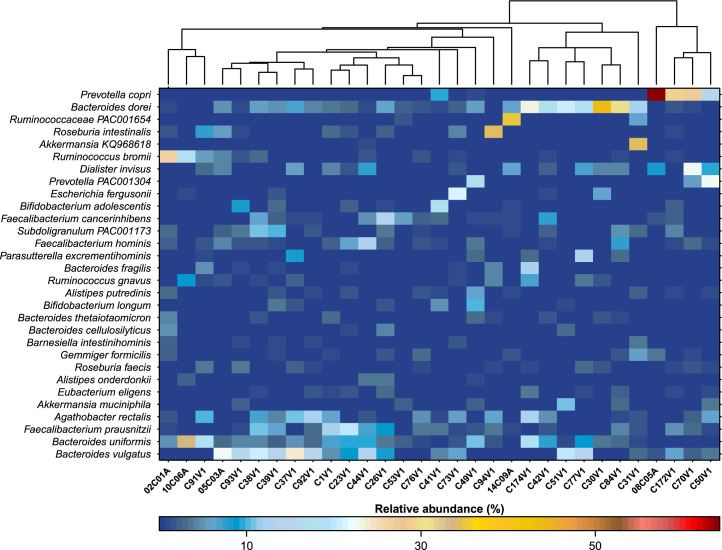
Intestinal microbial communities at inclusion in the study. 31 patients were analyzed for their microbial composition before treatment with 1,8-Cineol. The most abundant taxa classified at the species level are depicted in a heatmap based on their relative abundance per sample.

We investigated the gut microbial composition of 31 CRSwNP patients before and after two weeks of treatment with prescribed Soledum Kapseln forte (capsules 3 × 200 mg 1,8-Cineol/day). Overall, data revealed high abundances of various *Bacteroides* species (e. g. *B. dorei, B. uniformis, B. vulgatus*), *Faecalibacterium* species (e.g., *F. prausnitzii, F. hominis*), *Agathobacter rectalis, Dialister invisus* and other species (e.g., *Ruminococcus bromii* and *R. gnavus*) as part of the data set (Fig. 1). In some patients, *Prevotella copri* was the dominating species of the gut microbiota (Fig. 1). Supplementary Figure 1A depicts microbial abundances of the given species after 1,8-Cineol treatment of the same patients.

**Fig. 2:**
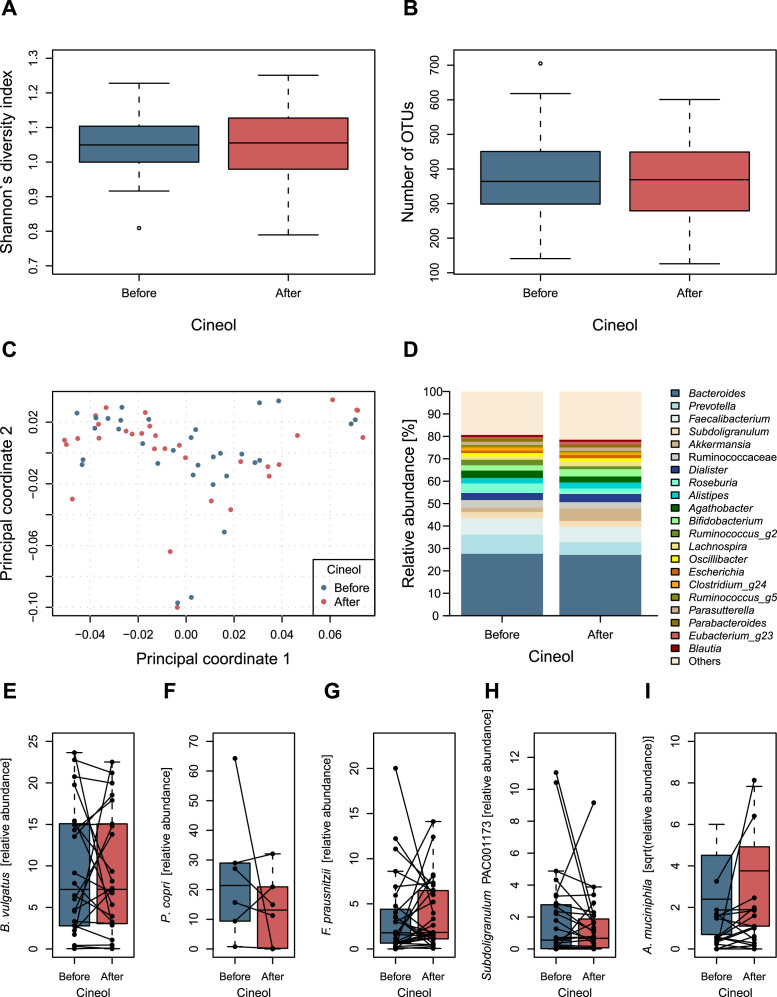
Standard metrics assessing microbial composition and diversity do not reveal differences originating from 1,8-Cineol treatment. Neither Shannon´s diversity index (A) nor number of observed OTUs (B) change upon 1,8-Cineol treatment. Also, Principal Coordinates Analysis (**C**) does not appear to display changes that would be attributable to 1,8-Cineol. Genus level assigment of bacterial taxa overall shows comparable mean abundance values before and after treatment with a slightly reduced abundance of the taxa *Prevotella* (**D**). Relative abundance of selected high abundant taxa classified to species level cannot depict significant changes for *Bacteroides vulgatus* (**E**), *Prevotella copri* (**F**), *Faecalibacterium prausnitzii* (**G**), and *Subdoligranulum* sp (**H**)*.* For *Akkermansia muciniphila* we observe an increase throughout 1,8-Cineol treatment being close to significance (*p* = 0.0571) when using Paired Wilcoxon rank-sum test (**I**).

To evaluate the impact of treatment on the gut microbiota, we compared different aspects of microbial composition and diversity before and after treatment with 1,8-Cineol. Shannon´s diversity index showed no differences when comparing alpha-diversity before and after treatment (Fig. 2A) and no shifts could be observed when assessing the number of observed OTUs (operational taxonomic units) (Fig. 2B) on a 97 % similarity threshold.

Principal Coordinates Analysis (Fig. 2C) depicts similar sample distribution before and after 1,8-Cineol treatment and a Constrained Correspondence Analysis depicts only non-significant shifts within the microbiota when using the variable before/after treatment with 1,8-Cineol as a constraint (Suppl. Fig. 2).

When assessing major taxa on genus level we observed comparable mean relative abundances for taxa such as *Bacteroides, Faecalibacterium, Delosigranulum* and others, while among the most prevalent genera, slightly reduced presence of the taxa *Prevotella* as well as a non-significant increase of *Akkermansia* can be observed (Wilcoxon rank-sum test; *p* = 0.1218) (Fig. 2D). Similar results are being depicted on the respective class level with Clostridia and Bacteroidia being the major taxa within this data set (Suppl. Fig. 3). We further depicted the most abundant species level assignments for the above-named genera. No significant changes could be observed when addressing patients presenting with *B. vulgatus, P. copri, F. prausnitzii, Delosigranulum* sp. PAC001173*,* and *A. muciniphila (*Fig. 2*E-I).* While *A. muciniphila* displays higher abundance values in most patients following 1,8-Cineol treatment, this change narrowly misses significance (Wilcoxon rank-sum test; *p* = 0.0571). When assessing associations of taxa to 1,8-Cineol treatment by the MaAsLin2 algorithm, associations could be shown neither on class- nor on genus or species level.

## Discussion

The present study was undertaken to investigate the influence of the anti-inflammatory natural monoterpene 1,8-Cineol on the intestinal microbial communities in patients suffering from the chronic inflammatory disease CRSwNP. Our interest in 1,8-Cineol stems from studies that demonstrated anti-microbial activities of 1,8-Cineol in different inflammatory and infectious diseases such as bronchial asthma, chronic rhinosinusitis or *Klebsiella pneumoniae* infections (Moo et al., 2021; Juergens et al., 2003; Bruchhage et al., 2018; Schurmann et al., 2019). Thus, recent investigations of ear swab samples from otitis media patients revealed reduced appearances of associated pro-inflammatory bacterial species *Klebsiella pneumoniae, Enterococcus cloacae, Pseudomonas aeruginosa, Proteus mirabilis*, and *Staphylococcus aureus* in response to 1,8-Cineol (CNL-1976®), which was administered as the clinically approved drug Soledum® Kapseln forte (capsules) (Leichtle et al., 2024).

The enteric coating of the orally applied 1,8-Cineol containing capsules ensures their passage through the stomach and allows the development of the anti-inflammatory effect via the gut and the bloodstream (MacKenzie et al., 2023). Hence, the gut microbial community, which is implicated in a wide range of inflammatory diseases, gets directly and immediately influenced upon 1,8-Cineol administration (Kociszewska and Vlajkovic, 2022; Vijay and Valdes, 2022). In the light of the potential anti-bacterial properties of 1,8-Cineol this must be given special attention because changes and disruptions of the gut microbial community have been closely linked to different inflammatory pathologies including inflammatory bowel disease (Nguyen et al., 2020), colorectal cancer (39) as well as other gastrointestinal infections (Lynch and Martinez, 2002; Kim et al., 2022; Johnson et al., 1999). Furthermore, gut microbiota alterations have been shown to affect systemic immune responses via the gut-associated lymphatic tissues (Pabst and Mowat, 2012). Thus, the regulation of gut microbial dynamics in response to antibiotic exposure and evaluating alternative treatment options are receiving increasing attention.

In this context it is particularly relevant that our data revealed no overall significant disruption of the gut microbial composition and diversity upon 1,8-Cineol treatment, but rather individual and patient-specific variations that were largely independent of 1,8-Cineol. We want to highlight that *Prevotella copri* was identified as a dominating bacterial species of the gut microbiota in certain patients, which has been associated with fiber-enriched nutrition as known for, e.g., vegetarians (Wu and Kasper, 2020). In this light we recently observed that successful treatment of patients with chronic otitis media using 1,8-Cineol is linked to the presence of *P. copri* (Leichtle et al., 2024). Given the results described here, it appears likely that the underlying effects of this observation are not due to support in the growth of *P. copri* but rather through functional interactions which may occur only in the presence of *P. copri*. Regarding the functional basis for treatment success in COM patients, it might be of interest that *P. copri* has been shown to directly influence metabolic pathways and immune responses via the production of branched-chained amino acids (BCAA) (Agus et al., 2021; Tajiri and Shimizu, 2018).

Of note, we observed a trend towards increased abundances of *Akkermansia muciniphila* (*p* = 0.0571) within our cohort of CRSwNP patients upon 14 days of 1,8-Cineol administration. *A. muciniphila* is a gram-negative anaerobic bacterium of the *Verrucomicrobia* phylum that colonizes the intestinal mucosa and comprises approximately 3 % of the total microbiota in healthy individuals (Derrien et al., 2008; Derrien et al., 2004). In the recent past, *A. muciniphila* has gained increasing attention as a promising probiotic bacterial species due to lower abundances in the gut of patients with autoimmune, metabolic, and chronic inflammatory diseases (Zhai et al., 2019; Everard et al., 2013; Pittayanon et al., 2020; Zheng et al., 2018). The abundance of *A. muciniphila* has been inversely correlated with a range of inflammatory diseases, such as inflammatory bowel disease, and diabetes in human as well as in mice (Pu et al., 2021; Pittayanon et al., 2020). Decreased presence of *A. muciniphila* has also recently been observed in patients with chronic rhinosinusitis (Michalik et al., 2023). Based on these observations, several studies evaluated the probiotic potential of *A. muciniphila* with regard to different inflammatory diseases, which revealed a better therapeutic effect of outer membrane compounds compared to living bacteria (Santacruz et al., 2010; Parks et al., 2013; Yassour et al., 2016; Kang et al., 2013; Ashrafian et al., 2021; Choi et al., 2021).

Taken together, this study supports the notion of 1,8-Cineol being a valuable treatment option for inflammatory diseases of the upper aerodigestive tract by highlighting that its treatment goes without profound disturbances of the gut microbial community and may even be supportive to pre- and probiotic interventions. Further investigations on larger CRS study cohorts over more extended periods will help to better understand the influence of 1,8-Cineol on the individual gut microbiota dynamics in relation to the course of this inflammatory disease, maybe also in view of a supporting personalized prebiotic and probiotic treatment.

## Ethics approval and consent to participate

All patients of the present study (approval number 18–322 from the ethics committee of the University of Luebeck) were examined and treated at the Department of Otorhinolaryngology, University Hospital Schleswig-Holstein, Campus Luebeck, after they have given their written informed consent.

## Funding

This research was funded by Cassella-med GmbH & Co. KG, Cologne, Germany.

## CRediT authorship contribution statement

**Simon Graspeuntner:** Conceptualization, Methodology, Investigation, Resources, Data curation, Writing – original draft, Writing – review & editing. **Mathias Heidemann:** Methodology, Investigation, Writing – review & editing. **Stephanie Jeschke:** Methodology, Investigation, Writing – review & editing. **Mariia Lupatsii:** Methodology, Investigation, Data curation, Writing – original draft, Writing – review & editing. **Zuzana Penxova:** Methodology, Investigation, Writing – review & editing. **Sven Künzel:** Methodology, Investigation, Data curation, Writing – review & editing. **Barbara Wollenberg:** Conceptualization, Writing – review & editing. **Anke Leichtle:** Writing – review & editing. **Michael Ploch:** Writing – review & editing. **Karl-Ludwig Bruchhage:** Resources, Writing – review & editing. **Ralph Pries:** Conceptualization, Resources, Data curation, Writing – original draft, Writing – review & editing.

## Declaration of competing interest

The authors declare the following financial interests/personal relationships which may be considered as potential competing interests: Ralph Pries, Karl-Ludwig Bruchhage, Anke Leichtle reports financial support was provided by Cassella-med GmbH & Co. KG, Cologne, Germany. Ralph Pries, Karl-Ludwig Bruchhage reports a relationship with Cassella-med-GmbH, Cologne, Germany that includes: funding grants. If there are other authors, they declare that they have no known competing financial interests or personal relationships that could have appeared to influence the work reported in this paper.

## Data Availability

Data is provided within the manuscript or supplementary information files. Sequencing data are available at the European Nucleotide Archive (ENA) under Accession Number PRJEB88961.

## Appendix Supplementary materials

Image, application 1
