# Supplementary material for: Gut microbial communities in chronic rhinosinusitis patients in response to 1,8-Cineol treatment
Source: Curr Res Microb Sci. 2025 Jul 22;9:100442. doi: 10.1016/j.crmicr.2025.100442 (PMC12332871; doi:10.1016/j.crmicr.2025.100442)
Supplement: Supplementary file 1 [file mmc1.docx]

Supplement


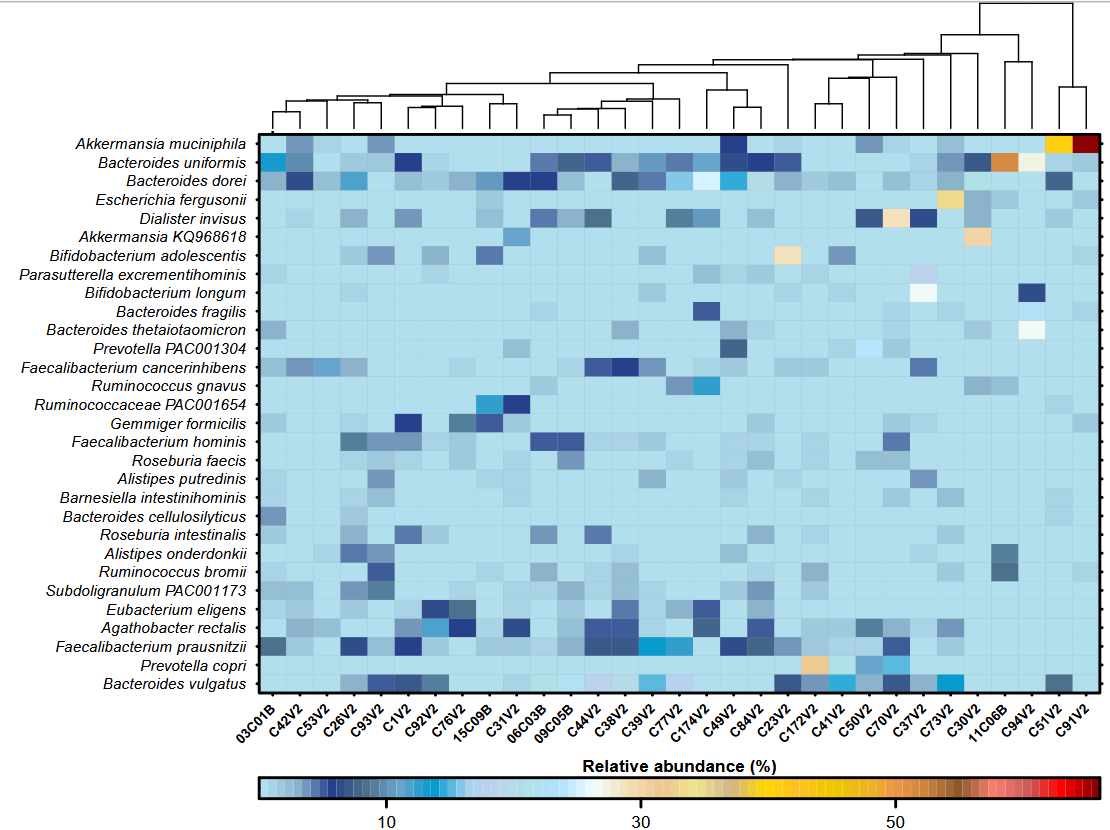


Supplementary Figure 1: Intestinal microbial communities after 14 days of 1,8-Cineol-treatment. The most abundant taxa classified at the species level are depicted in a heatmap based on their relative abundance per sample. Patients and microbial taxa are corresponding to Figure 1.


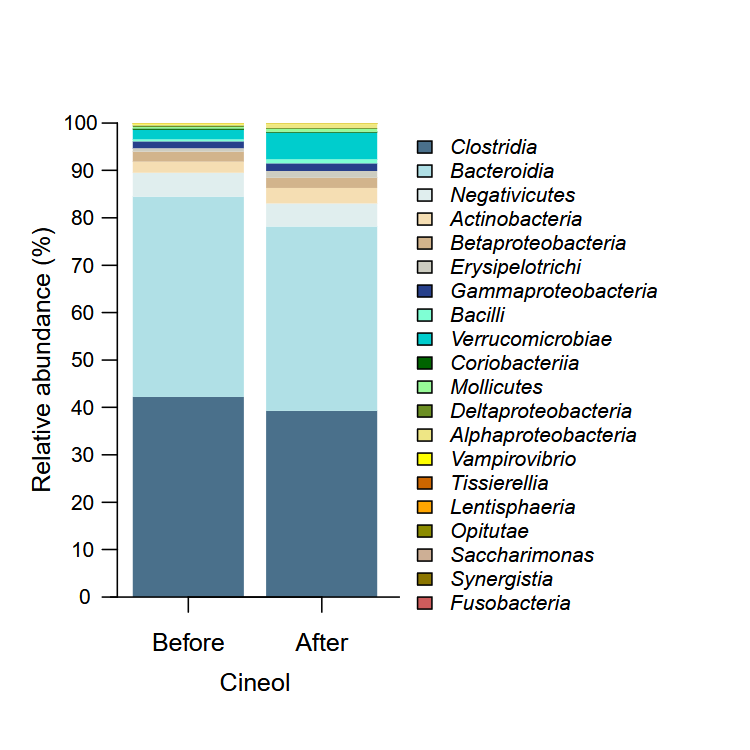


Supplementary Figure 2: Mean abundance values for class level microbial taxa. Assignment of bacterial taxa overall shows comparable mean abundance values before and after treatment


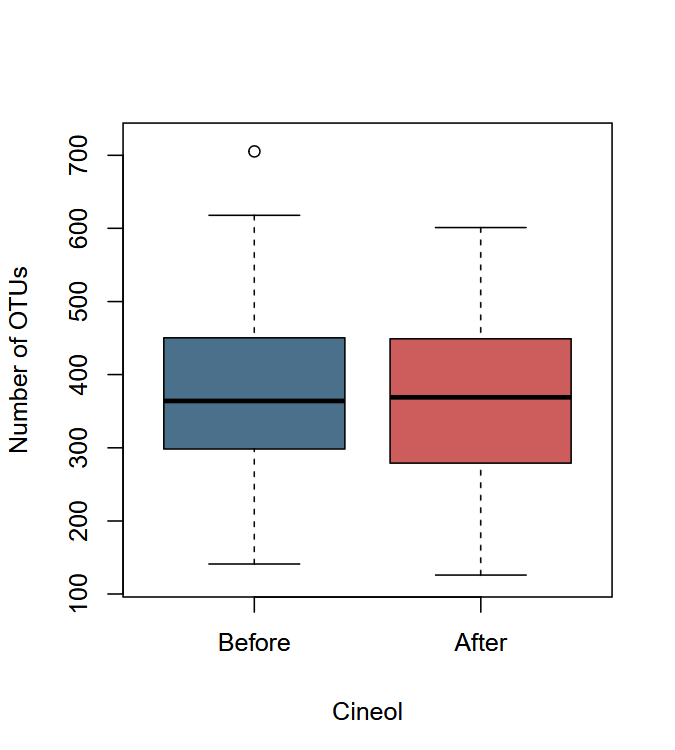

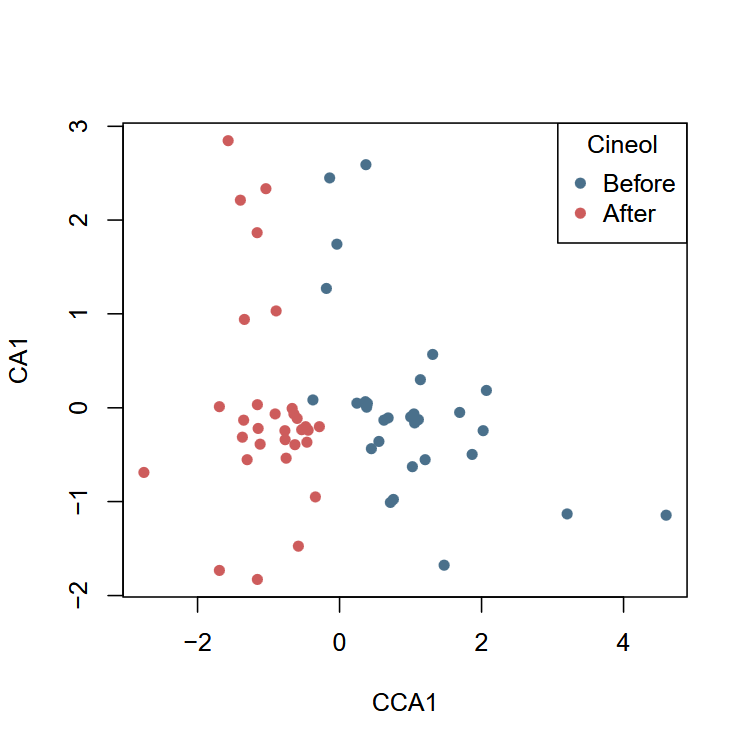


Supplementary Figure 3: Additional measures of alpha-diversity and beta-diversity depict no differences before and after treatment with 1,8-Cineol. The number of observed species is equal (A) and shifts in Constraint Corresponding analysis using before/after as a constraint (B) are non-significant (permutation test for CCA: *p=1.000*).
